# Global Observability Analysis of Rotational MEMS Inertial Navigation System for Land Vehicle Applications

**DOI:** 10.3390/s26092599

**Published:** 2026-04-23

**Authors:** Wenhui Yang, Xin Zhao, Jinhao Song, Yong Li

**Affiliations:** 1 School of Electronic Science and Engineering, University of Electronic Science and Technology of China, Chengdu 611731, China; 202311021912@std.uestc.edu.cn (W.Y.); 202411021631@std.uestc.edu.cn (J.S.); 2 Chengdu CAIC Electronic Co., Ltd., Chengdu 610091, China

**Keywords:** rotation scheme, global observability, sensor bias estimation, MEMS-INS

## Abstract

In GNSS-denied environments, Micro-Electromechanical Systems–Inertial Navigation Systems (MEMS-INS) play a critical role in sustaining the autonomous operation of vehicles. However, the inherent accuracy constraints of MEMS inertial sensors often hinder their broader application. To address this limitation, this paper proposes a novel integrated navigation system based on a rotating MEMS architecture. Through a global observability analysis, the proposed method enables the effective separation of sensor biases, mitigates long-term error accumulation, and significantly enhances the estimation accuracy of both attitude and velocity. Simulation results indicate that a rotation scheme around the X-axis allows for the reliable estimation of sensor biases. Moreover, road test results reveal that when the vehicle experiences variations in angular velocity, the rotating configuration consistently outperforms its fixed counterpart, reducing heading, position, and velocity errors by 75.3%, 62.9%, and 76.0%, respectively.

## 1. Introduction

The development of autonomous vehicles as a fundamental component of smart city transportation systems is expected to significantly increase the demand for high-precision positioning services. Because of its compact size, light weight, and affordability, the GNSS/MEMS INS integrated navigation system has greatly advanced autonomous vehicles across numerous smart city applications [1,2]. However, challenges such as GNSS signal blockage [3] and inherent MEMS issues—including significant bias instability and high noise levels—often lead to navigation drift. To address these challenges, complementary sensors like Lidar, Radar, and vision-based systems [4,5,6,7,8,9,10,11] have been employed to mitigate error accumulation. However, these solutions often involve high operational costs and remain susceptible to environmental factors. Consequently, there is renewed motivation to refocus on enhancing the intrinsic capabilities of self-contained inertial navigation systems to achieve robust and cost-effective positioning.

Furthermore, rotating an IMU at a fixed speed can modulate constant inertial sensor biases into sinusoidal signals that are canceled after integration over a rotation period [12,13]. While earlier systems leveraged the superior bias stability of Fiber-Optic Gyroscopes (FOGs) [14], recent advancements in MEMS technology [15] have made lightweight, cost-effective MEMS-based rotational mechanisms a viable alternative. Previous studies, such as those by Collin et al. [16,17,18] and X. Niu et al. [19], explored wheel-mounted rotational systems for dead reckoning. However, these configurations frequently rely on the assumption of horizontal planar motion, as they cannot directly capture the vehicle’s pitch information. To overcome this limitation, this article proposes an independent uniform rotation mechanism. Unlike wheel-mounted designs, this architecture flexibly adapts to diverse road conditions by effectively retaining key pitch dynamics, thereby enhancing the system’s capability to mitigate dynamic errors in complex environments.

System performance is fundamentally constrained by state observability. Traditional integrated navigation systems often exhibit limited accuracy due to the poor observability of key states, such as sensor biases. Conventional analysis methods, such as Piecewise Constant Systems (PWCSs) and Singular Value Decomposition (SVD) [20,21,22], primarily rely on linearization. However, these linearized insights may not fully reflect the intrinsic properties of the original nonlinear systems [23]. To overcome this, global observability analysis [24] has been utilized in various navigation contexts [25,26,27,28]. Yet, its application in defining the theoretical boundaries of rotational MEMS architectures—specifically for sensor bias separation under diverse vehicle dynamics—remains insufficiently explored.

This study proposes three MEMS IMU-based rotation schemes to assist integrated navigation systems, with a global observability analysis conducted to elucidate the conditions for sensor bias separation. The main contributions are summarized as follows:

1. A novel independent uniform rotation mechanism is proposed to enable real-time motion decoupling between the sensor and vehicle coordinate frames, significantly enhancing navigational stability and estimation reliability.

2. A comprehensive state estimation framework grounded in global observability theory is established to systematically evaluate the efficacy of various rotation strategies in decoupling sensor biases.

## 2. Dynamical Model of Rotary Ins

**Figure 1:**
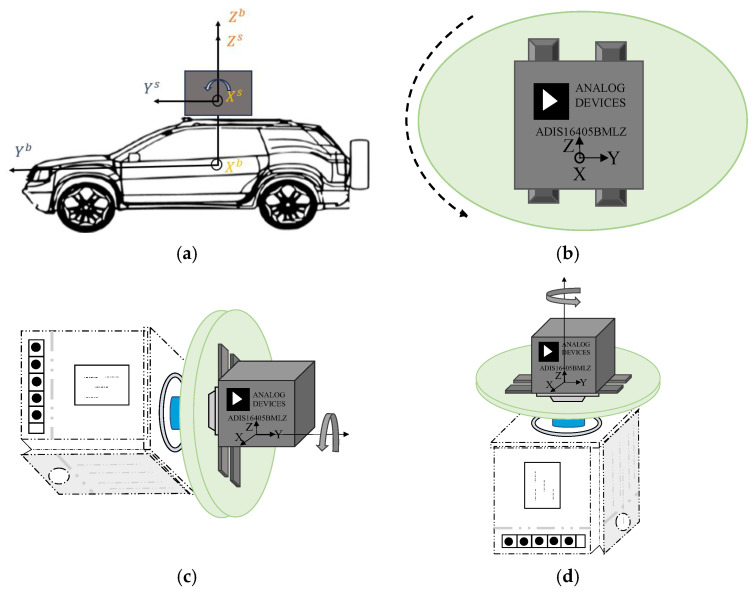
Schematics of rotary platform mounting configurations and three rotation schemes. (**a**) Navigation system for vehicles based on the rotating MIMU. (**b**) Rotating around sensor’s X-axis. (**c**) Rotating around sensor’s Y-axis. (**d**) Rotating around sensor’s Z-axis.

**Figure 2:**
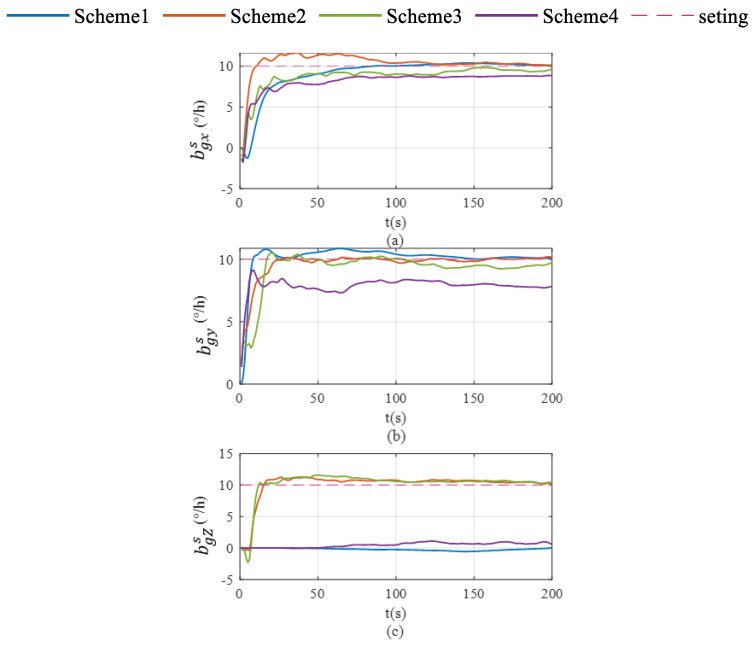
Gyroscope bias estimation in single-axis rotation under vehicle stationary conditions. (**a**) gyroscope bias estimation for the X-axis; (**b**) gyroscope bias estimation for the Y-axis; (**c**) gyroscope bias estimation for the Z-axis.

**Figure 3:**
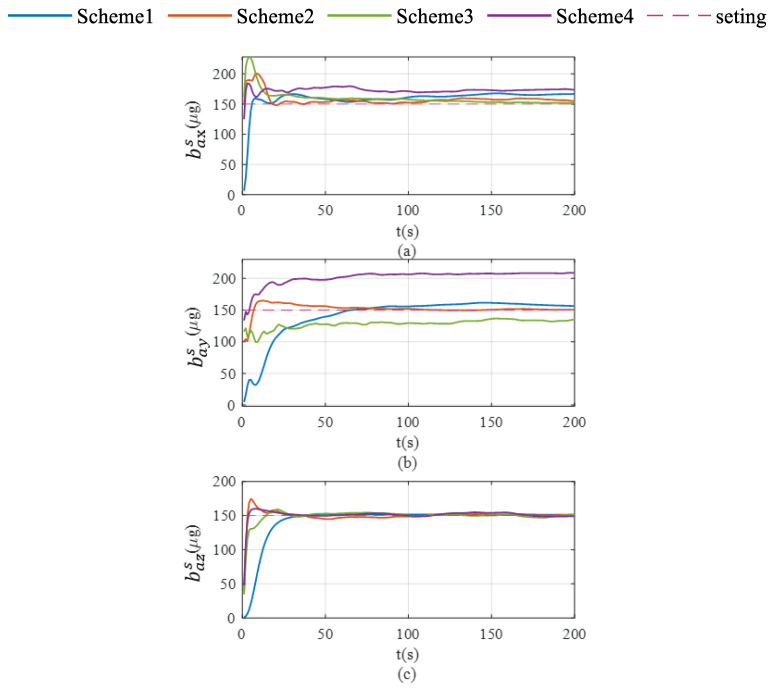
Accelerate bias estimation in single-axis rotation under vehicle stationary conditions. (**a**) accelerate bias estimation for the X-axis; (**b**) accelerate bias estimation for the Y-axis; (**c**) acceleration bias estimation for the Z-axis.

This section details three rotation schemes and establishes a rotation-based dynamic model, utilizing the traditional dynamic model as its foundation. The coordinate system of the rotational INS, with the rotary platform mounted on the vehicle, is illustrated in Figure 1a. Prior to rotation, the sensor’s Y-axis is aligned with the vehicle’s heading, the X-axis points to the vehicle’s right, and the Z-axis completes a right-handed Cartesian coordinate system. Based on this initial configuration, three distinct rotation schemes are investigated: (1) rotation about the X-axis (Figure 1b); (2) rotation about the Y-axis (Figure 1c); (3) rotation about the Z-axis (Figure 1d). The proposed navigation scheme employs a constant-rate rotary mechanism to acquire dynamic sensor data. Through real-time coordinate transformations, the rotational-state measurements are converted into equivalent static-state parameters. By decoupling the IMU frame from the vehicle body frame, this procedure accurately reconstructs the vehicle’s kinematic trajectory, yielding results that are fully compatible with standard navigation algorithms. Without loss of generality, the local-level frame (East–North–Up) is selected as the navigation reference frame, denoted as N. Furthermore, the vehicle body frame is denoted as B, the Earth frame as E, the inertial frame as I, and the IMU sensor frame as S.

The body attitude matrix with respect to the reference frame is $C_{b}^{n}$, the ground velocity is $v^{n}={\left[\begin{array}{ccc} v_{E} & v_{N} & v_{U} \end{array}\right]}^{T}$, and the dot above the variable denotes its first-order time derivative. The corresponding kinematic equations for the INS based on a fixed IMU are given as

(1) $${\dot{C}}_{b}^{n}=C_{b}^{n}(\omega_{nb}^{b}\times )$$

(2) $$\;\omega_{nb}^{b}=\omega_{ib}^{b}-b_{g}^{b}-C_{n}^{b}(\omega_{ie}^{n}+\omega_{en}^{n})$$

(3) $${\dot{v}}^{n}=C_{b}^{n}(f^{b}-b_{a}^{b})-(2\omega_{ie}^{n}+\omega_{en}^{n})\times v^{n}+g^{n}$$

where $\omega_{nb}^{b}$ is the body angular rate with respect to the reference frame, expressed in the body frame (B-frame); $\omega_{ib}^{b}$ is the error-contaminated body angular rate measured by gyroscopes in the B-frame; $\omega_{ie}^{n}={\left[0,\omega_{ie}\cos L,\omega_{ie}\sin L\right]}^{T}$ and $(2\omega_{ie}^{n}+\omega_{en}^{n})\times v^{n}$ are the Earth rotation rate in the reference frame, with $\omega_{ie}$ being the Earth rotation rate about its axis and L being the local latitude; $\omega_{en}^{n}={\left[-\frac{v_{N}}{R_{M}+h},\frac{v_{E}}{R_{N}+h},\frac{v_{E}\tan L}{R_{N}+h}\right]}^{T}$ is the angular rate of the reference frame with respect to the Earth frame, expressed in the navigation frame (N-frame); $R_{E}$ and $R_{N}$ are, respectively, the transverse radius and the meridian radius of curvature; h is the altitude; $f^{b}$ is the error-contaminated specific force measured by accelerometers in the B-frame; $b_{a}^{b}$ and $b_{g}^{b}$ represent the accelerometer and gyroscope biases both resolved in the B-frame; $g^{n}=[\begin{array}{ccc} 0 & 0 & -g \end{array}{]}^{T}$ is the gravity vector in the N-frame, and g is the magnitude of local gravity. (⋅×) represents the antisymmetric matrix of the vector.

The gyroscope bias $b_{g}^{b}$ and accelerometer bias $b_{a}^{b}$ resolved in the B-frame are approximated as random constant vectors, where 0 represents the zero vector.

(4) $${\dot{b}}_{g}^{b}=0,\;{\dot{b}}_{a}^{b}=0$$

assuming the vehicle operates at low speeds (<10 m/s) so that the terms $\omega_{en}^{n}$ in Equation (1) and $(2\omega_{ie}^{n}+\omega_{en}^{n})\times v^{n}$ in Equation (2) are both negligible. This study constrains the magnitudes of parameters $\omega_{en}^{n}$ and $(2\omega_{ie}^{n}+\omega_{en}^{n})\times v^{n}$ to 10^−6^ and 10^−4^, respectively, with negligible effects on other system states. Building on this framework, article [29] adopts the same simplification approach to reduce computational load while meeting real-time requirements.

(5) $$\omega_{nb}^{b}=\omega_{ib}^{b}-b_{g}^{b}-C_{b}^{n}\omega_{ie}^{n}$$

(6) $${\dot{v}}^{n}=C_{b}^{n}(f^{b}-b_{a}^{b})+g^{n}$$

Furthermore, adding a rotating sensor frame S, the above Equations (1) and (4)–(6) can be re-written as

(7) $${\dot{C}}_{b}^{s}=C_{b}^{s}(\omega_{sb}^{s}\times )$$

(8) $$\omega_{nb}^{b}=C_{s}^{b}(\omega_{is}^{s}-b_{g}^{s})-C_{n}^{b}\omega_{ie}^{n}$$

(9) $${\dot{v}}^{n}=C_{b}^{n}C_{s}^{b}(f^{s}-b_{a}^{s})+g^{n}$$

(10) $${\dot{b}}_{g}^{s}=0,\;{\dot{b}}_{a}^{s}=0$$

where $\omega_{is}^{s}$ is the error-contaminated body angular rate measured by gyroscopes in the S-frame, $b_{a}^{s}$ and $b_{g}^{s}$ represent the accelerometer and gyroscope biases both resolved in the S-frame, and $f^{s}$ is the error-contaminated specific force measured by the accelerometer in the S-frame.

At the initial operation of the system, it is assumed that the B-frame and S-frame are aligned. The orientation matrix of the sensor relative to the vehicle $C_{s}^{b}$ at any given moment is known, which is due to the constant rotation of the motor.

## 3. Global Perspective Observability

This section develops a mathematical model for the INS utilizing a rotating MEMS IMU, with comprehensive global observability analysis conducted under varying motion scenarios. It is important to emphasize that the ‘global observability’ analyzed in this section mathematically refers to the exact evaluation of the original nonlinear system over a finite time interval, distinguishing it from local linearization methods. This rigorous mathematical framework is established under specific physical kinematic assumptions (e.g., low-speed constraints), which define the practical boundary conditions for the subsequent performance evaluations.

**Lemma** **1.** *For any two linearly independent vectors, if their coordinates in two arbitrary frames are given, then the attitude matrix between the two frames can be determined.*

**Lemma** **2** ([27])**.** *Let* a *and* b *be known three-dimensional vectors satisfying* a×m=b(a≠0)*, where* m *is an unknown vector: If* |m| *is given, then* m *has solutions expressed as* $m=\pm a\sqrt{|a{|}^{2}|m{|}^{2}-|b{|}^{2}}/|a{|}^{2}-a\times b/|a{|}^{2}$*.*

**Lemma** **3.** *Let* A *and* B *be two vectors. The magnitude squared of the cross product of these two vectors,* A×B*, is given by* $|A\times B{|}^{2}=|A{|}^{2}|B{|}^{2}-{\left(A\cdot B\right)}^{2}$*.*

**Lemma** **4.** *Given two vectors* a *and* b*, their cross product* a×b *is a new vector that is perpendicular to the plane spanned by* a *and* b*.*

**Lemma** **5.** *Let* a *and* b *be two vectors. The magnitude squared of the cross product of these two vectors,* a×(a×b)*, is given by* $a\times (a\times b)=a\cdot (a\cdot b)-b\cdot {\left|a\right|}^{2}$*.*

Designate an I-frame to be the N-frame at t=0, i.e., I=N(0). Decompose the body attitude matrix at the current time as

(11) $$C_{n}^{b}=C_{b(0)}^{b(t)}C_{n(0)}^{b(0)}C_{n(t)}^{n(0)}=C_{b(0)}^{b(t)}C_{n}^{b}(0)C_{n(t)}^{n(0)}$$

where $C_{b(0)}^{b(t)}$ and $C_{n(t)}^{n(0)}$ are attitude matrices operating as functions of $\omega_{ib}^{b}-b_{g}^{b}$ and $\omega_{ie}^{n}$, respectively. They encode, respectively, the attitude changes in the B-frame and the N-frame from time 0 to t. Substituting (9) gives

(12) $$C_{b(t)}^{b(0)}C_{s}^{b}(b_{a}^{s}-f^{s})=C_{n}^{b}\left(0\right)C_{n(t)}^{n(0)}g^{n}$$

The quantity $C_{n(t)}^{n(0)}g^{n}$ is the gravity vector seen from the I-frame, and its trajectory history forms a cone at all locations except the two Earth poles where the cone degenerates to a line. So there always exist two time instants that $C_{n(t)}^{n(0)}g^{n}$ has linearly independent directions. Using Lemma 1, the initial body attitude $C_{n}^{b}\left(0\right)$ can be solved if only $b_{a}^{s}$ and $b_{g}^{s}$ are given [26]. It must be emphasized that Equation (12) can be obtained from Equation (9) only when at rest or moving at a uniform speed.

### 3.1. Global Observability Analysis of IMU Single-Axis Rotation Navigation Scheme

When the MEMS IMU rotates at a constant velocity around a single axis while the vehicle remains stationary or performs uniform linear motion, $\omega_{nb}^{b}=0$ and ${\dot{v}}^{n}=0$. The motor rotation angle provides the $C_{b}^{s}$, and $\omega_{sb}^{b}$, the rotation vector of the IMU relative to the vehicle body, is a known quantity.

Rewrite Equations (8) and (9) as

(13) $$\omega_{is}^{s}-b_{g}^{s}=C_{n}^{s}\omega_{ie}^{n}$$

(14) $$0=C_{s}^{n}(f^{s}-b_{a}^{s})+g^{n}$$

Taking the time derivative of both sides of (13),

(15) $$0={\dot{\omega }}_{is}^{s}+(\omega_{ns}^{s}\times )C_{n}^{s}\omega_{ie}^{n}$$

Substituting the derived expression back into Equation (13),

(16) $${\dot{\omega }}_{is}^{s}=(\omega_{ns}^{s}\times )(b_{g}^{s}-\omega_{is}^{s})$$

Taking the time derivative of both sides of Equation (16),

(17) $$0=(\omega_{ns}^{s}\times ){\dot{\omega }}_{ns}^{s}+{\ddot{\omega }}_{is}^{s}$$

In addition, taking the time derivative of both sides of (14),

(18) $${\dot{f}}^{s}=(\omega_{ns}^{s}\times )(b_{a}^{s}-f^{s})$$

Taking the derivative of Equation (18) again,

(19) $$0=(\omega_{ns}^{s}\times ){\dot{f}}^{s}+{\ddot{f}}^{s}$$

To ensure a unique solution of $\omega_{sn}^{n}$, the directions of ${\dot{\omega }}_{is}^{s}$ and ${\ddot{\omega }}_{is}^{s}$, as well as ${\dot{f}}^{s}$ and ${\ddot{f}}^{s}$, must be non-collinear. Combining Lemma 5 with Equations (16) and (18), and utilizing the orthogonality of the 3D vector cross product (i.e., the cross product is strictly perpendicular to its operand vectors), the following expression can be derived:

(20) $$\begin{array}{cc} {\dot{f}}^{s}\times {\ddot{f}}^{s} & ={\dot{f}}^{s}\times ({\dot{f}}^{s}\times \omega_{ns}^{s})\\ & =-{\left|{\dot{f}}^{s}\right|}^{2}\omega_{ns}^{s}\\ & =-{\left|\omega_{ns}^{s}\times g^{n}\right|}^{2}\omega_{ns}^{s}\neq 0 \end{array}$$

(21) $$\begin{array}{cc} {\dot{\omega }}_{is}^{s}\times {\ddot{\omega }}_{is}^{s} & ={\dot{\omega }}_{is}^{s}\times ({\dot{\omega }}_{is}^{s}\times \omega_{ns}^{s})\\ & =-{\left|{\dot{\omega }}_{is}^{s}\right|}^{2}\omega_{ns}^{s}\neq 0 \end{array}$$

According to (16) and (18), using Lemma 2, attitude transformation does not change; thus, the solutions of $b_{a}^{s}$ and $b_{g}^{s}$, respectively, are

(22) $$b_{g}^{s}-\omega_{is}^{s}=\pm \frac{\omega_{ns}^{s}{\left({\left|\omega_{ns}^{s}\right|}^{2}{\left|b_{g}^{s}-\omega_{is}^{s}\right|}^{2}-{\left|-{\dot{\omega }}_{is}^{s}\right|}^{2}\right)}^{1/2}}{{\left|\omega_{ns}^{s}\right|}^{2}}-\frac{\omega_{ns}^{s}\times (-{\dot{\omega }}_{is}^{s})}{{\left|\omega_{ns}^{s}\right|}^{2}}$$

(23) $$b_{a}^{s}-f^{s}=\frac{\pm \omega_{ns}^{s}{\left\{{\left[\omega_{sn}^{n}\cdot (b_{a}^{s}-f^{s})\right]}^{2}\right\}}^{1/2}}{{\left|\omega_{sn}^{n}\right|}^{2}}-\frac{\omega_{ns}^{s}\times \{\omega_{sn}^{s}\times (b_{a}^{s}-f^{s})\}}{{\left|\omega_{ns}^{s}\right|}^{2}}$$

Using Lemmas 3 and 5, they are reduced to

(24) $$b_{g+,-}^{s}=\frac{\omega_{ns}^{s}\cdot [\omega_{ns}^{s}\cdot (C_{n}^{s}\omega_{ie}^{n})]}{{\left|\omega_{ns}^{n}\right|}^{2}}-\frac{\pm \omega_{ns}^{s}|\omega_{ns}^{s}\cdot (C_{n}^{s}\omega_{ie}^{n})|}{{\left|\omega_{ns}^{s}\right|}^{2}}+b_{g}^{s}$$

(25) $$b_{a,+,-}^{s}=\pm \frac{\omega_{ns}^{s}|\omega_{ns}^{s}\cdot (C_{n}^{s}g^{n})|}{{\left|\omega_{ns}^{n}\right|}^{2}}-\frac{\omega_{ns}^{s}\cdot [(C_{n}^{s}g^{n})\cdot \omega_{ns}^{s}]}{{\left|\omega_{ns}^{s}\right|}^{2}}+b_{a}^{s}$$

Here $\omega_{ns}^{s}=\omega_{nb}^{s}+\omega_{bs}^{s}$. However, under conditions of uniform linear motion or stationary status, $\omega_{ns}^{s}=\omega_{bs}^{s}$. Therefore, the aforementioned equation is simplified as

(26) $$b_{g+,-}^{s}=\frac{\omega_{bs}^{s}\cdot \left[\omega_{bs}^{s}\cdot (C_{n}^{s}\omega_{ie}^{n})\right]}{{\left|\omega_{bs}^{s}\right|}^{2}}-\frac{\pm \omega_{bs}^{s}|\omega_{bs}^{s}\cdot (C_{n}^{s}\omega_{ie}^{n})|}{{\left|\omega_{bs}^{s}\right|}^{2}}+b_{g}^{s}$$

(27) $$b_{a,+,-}^{s}=\frac{\pm \omega_{bs}^{s}\left|\omega_{bs}^{s}\cdot (C_{n}^{s}g^{n})\right|}{{\left|\omega_{bs}^{s}\right|}^{2}}-\frac{\omega_{bs}^{s}\cdot \left[(C_{n}^{s}g^{n})\cdot \omega_{bs}^{s}\right]}{{\left|\omega_{bs}^{s}\right|}^{2}}+b_{a}^{s}$$

Finally, when $\omega_{bs}^{s}$ is perpendicular to both $C_{n}^{s}\omega_{ie}^{n}$ and $C_{n}^{s}g^{n}$, both $b_{a}^{s}$ and $b_{g}^{s}$ have one solution. The observation of attitude can only be achieved when $b_{a}^{s}$ and $b_{g}^{s}$ can be uniquely determined. The above analysis indicates that under specific geographical conditions, if the projection of the gravity vector and the Earth’s rotation vector are coplanar, and the rotation axis oriented around the X-axis is uniquely perpendicular to that plane, the separation of sensor deviations can be achieved. However, due to strict coplanar conditions, this ideal situation is difficult to achieve in practice. Therefore, it is necessary to conduct a systematic global observability analysis of the rotation scheme for moving vehicles.

### 3.2. Global Observability Analysis of IMU Single-Axis Rotation and Vehicle Mobility Scheme

Furthermore, specific conditions under which sensor biases are decoupled during vehicle motion are derived. In land-based applications, particularly when undergoing uniform circular motion, wheeled vehicles do not operate in unrestricted three-dimensional space. Instead, they are subject to non-holonomic constraints (NHCs), which dictate that the velocity components in the plane perpendicular to the forward direction of motion are zero. Under these non-holonomic constraints, the measurement equation is formulated as

(28) $$y=v^{b}=C_{n}^{b}v^{n}$$

where $v^{b}$ denotes the vehicle velocity. Taking the time derivative of both sides of (28) and substituting into Equation (9),

(29) $$C_{b}^{n}(\omega_{nb}^{b}\times )y+C_{b}^{n}\dot{y}=C_{s}^{n}(f^{s}-b_{a}^{s})+g^{n}$$

When the vehicle with a fixed IMU is performing low-speed uniform circular motion, the velocity vector in the body frame remains consistently aligned with the vehicle’s forward axis while maintaining constant magnitude, yielding $\dot{y}=0$ $\omega_{nb}^{b}\neq 0$ and ${\dot{\omega }}_{nb}^{b}=0$.

(30) $$\omega_{nb}^{b}=\omega_{ib}^{b}-b_{g}^{b}-C_{n}^{b}\omega_{ie}^{n}$$

(31) $$C_{b}^{n}(\omega_{nb}^{b}\times )y=C_{b}^{n}(f^{b}-b_{a}^{b})+g^{n}$$

Taking the time derivative of the above formula,

(32) $$0={\dot{\omega }}_{ib}^{b}+(\omega_{nb}^{b}\times )(\omega_{ib}^{b}-b_{g}^{b})$$

(33) $$(\omega_{nb}^{b}\times )(\omega_{nb}^{b}\times )y=(\omega_{nb}^{b}\times )(f^{b}-b_{a}^{b})+{\dot{f}}^{b}$$

Taking the second derivative of the above expression,

(34) $$0={\ddot{\omega }}_{ib}^{b}+\omega_{nb}^{b}\times {\dot{\omega }}_{ib}^{b}$$

(35) $$0={\ddot{f}}^{b}+\omega_{nb}^{b}\times {\dot{f}}^{b}$$

In uniform circular motion, the directions of ${\dot{\omega }}_{ib}^{b}$ and ${\dot{f}}^{b}$ are changing, so $\omega_{nb}^{b}$ can be uniquely determined.

When the vehicle executes uniform circular motion with the IMU rotating uniformly about a single axis, differentiate Equations (8) and (29).

(36) $$\omega_{ns}^{s}\times b_{g}^{s}=\omega_{ns}^{s}\times \omega_{is}^{s}+{\dot{\omega }}_{is}^{s}$$

(37) $$\omega_{ns}^{s}\times b_{a}^{s}=\omega_{ns}^{s}\times f^{s}+{\dot{f}}^{s}-C_{b}^{s}(\omega_{nb}^{b}\times )(\omega_{nb}^{b}\times )y$$

Given that $\omega_{ns}^{s}=\omega_{nb}^{s}+\omega_{bs}^{s}$ and $\omega_{nb}^{s}=C_{b}^{s}\omega_{nb}^{b}$ are known quantities, and considering the vehicle’s uniform circular motion ensures vector independence across sub-intervals, $b_{g}^{s}$ and $b_{a}^{s}$ can be uniquely solved. However, simultaneous rotation about the Y-axis and the vehicle’s circular motion (rotation about the Z-axis) induces linear dependence among the columns. This rank deficiency precludes unique solutions, thereby compromising subsequent state discrimination of biases.

**Definition** **1** ([30])**.** *If for any unknown initial state* $x(t_{0})$*, there exists* $t_{1}>t_{0}$ *such that the system inputs and outputs over the time interval* $\left[t_{0},t_{1}\right]$ *can uniquely determine the initial state* $x(t_{0})$*, then the system is said to be observable.*

For the integrated system, states to be estimated include position, velocity, gyroscope bias, and accelerometer bias. Available information consists of the specific force recorded by the accelerometer and the body angular rate recorded by the gyroscope. By definition, the system is considered observable if the initial states can be uniquely determined from the measurements over a finite time interval.

While theoretical and experimental results strongly demonstrate the superiority of the X-axis rotation scheme, it must be clarified that the global observability analysis in Section 3 relies on several simplifying assumptions. These include neglecting the Earth’s rotation and transport rate terms based on a low-speed constraint (<10 m/s), assuming uniform circular motion, and applying idealized NHCs. These assumptions are well-suited for typical urban vehicles operating at low to moderate speeds. However, in high-speed scenarios or under severe road conditions (e.g., wheel slippage that violates the idealized NHCs), the applicability of these theoretical conclusions will be limited.

Under more general maneuvering conditions, characterized by continuous and random variations in acceleration and turning rates, it is difficult to provide a rigorous analytical proof within the current framework. Nevertheless, the rich dynamic excitations introduced by such complex maneuvers generally contribute to enhancing the practical observability of the system.

## 4. Simulation

In this section, raw accelerometer and gyroscope data are simulated under different IMU rotation modes for two scenarios: when the vehicle is stationary and when it is moving in uniform circular motion. Zero-speed information is introduced as observation information when the vehicle is stationary, while forward velocity information is incorporated as an observation when the vehicle is in uniform circular motion. Finally, the performance of different rotation algorithms is compared in terms of parameter estimation accuracy and error convergence characteristics.

### 4.1. IMU Single-Axis Rotation Navigation Scheme

In the conducted simulation, a vehicle is equipped with a navigation-grade IMU, which consists of a triad of gyroscopes (bias, 10°/h; random walks, 0.08°/$\sqrt{h}$) and accelerometers (bias, 150 µg; random walks, 10.78 ug/$\sqrt{\mathrm{Hz}}$). Output frequency is 100 Hz. The INS initial true attitude is set as ψ = 0°, φ = 0°, and θ =0°.

### 4.2. Scheme Design

The simulation process includes two major components: simulation execution and error analysis. The PSINS platform (http://psins.org.cn, accessed on 20 April 2026) was used for the simulation’s trajectory creation and IMU error modeling. The Kalman filter was updated using data on forward speed and zero velocity.

Simulations were conducted for seven different scenarios shown in Table 1 and Table 2. The single-axis rotation system aligns IMU’s XYZ axes with the East–North–Up (ENU).

(1) Single-Axis Rotation Scheme

The integrated navigation system simulation test lasts 200 s and the vehicle remains stationary.

**Figure 4:**
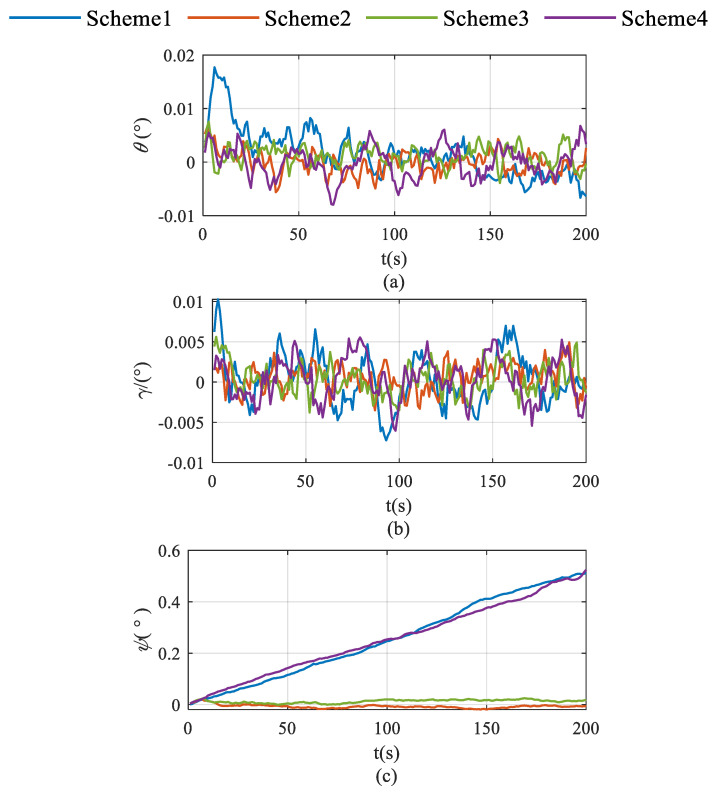
Roll, pitch and heading error accumulation characteristics in single-axis rotation under vehicle stationary condition. (**a**) for roll; (**b**) for pitch; (**c**) for heading.

**Figure 5:**
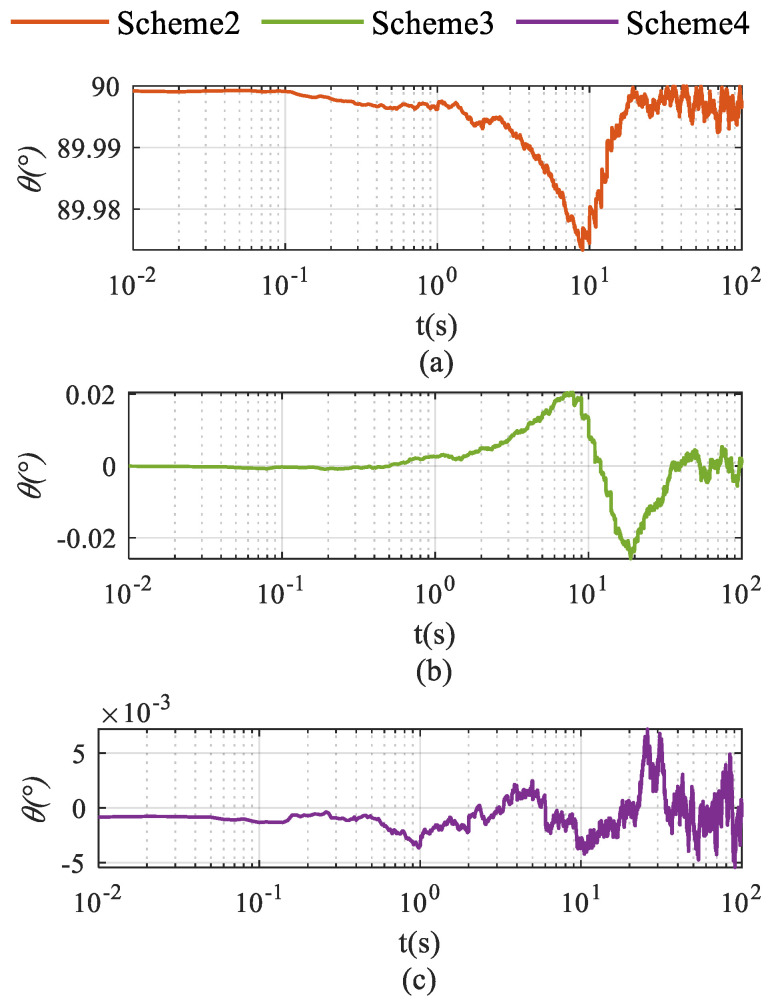
Vector angles for three schemes: (**a**) for Scheme 2; (**b**) for Scheme 3; (**c**) for Scheme 4.

**Figure 6:**
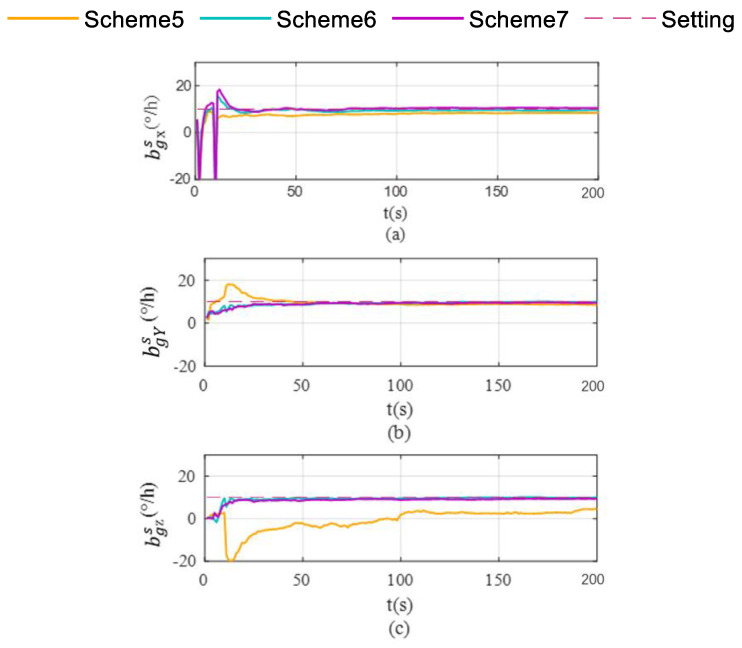
Gyroscope bias estimation in single-axis rotation with vehicle mobility condition. (**a**) gyroscope bias estimation for the X-axis; (**b**) gyroscope bias estimation for the Y-axis; (**c**) gyroscope bias estimation for the Z-axis.

Schemes were compared in terms of accelerometer bias estimation, gyroscope bias estimation, and the velocity and attitude errors shown in Figure 2, Figure 3, Figure 4, Figure 5 and Figure 6. As illustrated in Figure 2a, Scheme 1 fails to correct the gyroscope bias in the Z-axis. In contrast, Figure 2b,c demonstrate that both Scheme 2 and Scheme 3 significantly improve the correction of Z-axis gyroscope bias, with estimated values approaching the setting value. However, Figure 2c indicates that Scheme 4 exhibits minimal correction effectiveness for this parameter.

To systematically evaluate the proposed schemes, the comparative test schemes designed for vehicle stationary conditions are detailed in Table 1. Subsequently, the corresponding experimental configurations conducted under vehicle mobility conditions are outlined in Table 2 to validate the system’s dynamic performance.

As shown in Figure 3, Scheme 1 demonstrates effective correction of triaxial accelerometer biases. Under Scheme 2, these parameters exhibit significantly faster convergence rates and markedly reduced fluctuation ranges. In contrast, the other two rotation schemes show inferior performance in triaxial accelerometer bias estimation. Specifically, Scheme 3 displays evident deviations from setting values in X- and Y-axes’ accelerometer bias estimates, accompanied by increased fluctuation amplitudes. Similar deficiencies are observed in Scheme 4.

The accumulation of pitch and roll errors shows little variation across the four schemes, as illustrated in Figure 4. In contrast, both Scheme 2 and Scheme 3 significantly suppress heading error accumulation, with Scheme 2 demonstrating superior performance in this regard.

Figure 5 illustrates the angle between vector $\omega_{sb}^{s}$ and the plane spanned by vectors $\omega_{ie}^{s}$ and $g^{s}$. The results demonstrate that the angular parameter of Scheme 2 remains consistently stable at approximately 90°, whereas the angles of Scheme 3 and Scheme 4 fluctuate near 0°.

The simulation results demonstrate that Scheme 2 not only fully complies with the theoretical expectations of global observability, but also achieves higher-accuracy parameter estimation.

(2) Single-Axis Rotation with Vehicle Mobility Scheme

The integrated navigation system simulation test lasts 200 s and the maneuvers of uniform circular motion and linear acceleration are carried out to meet experimental requirements.

**Figure 7:**
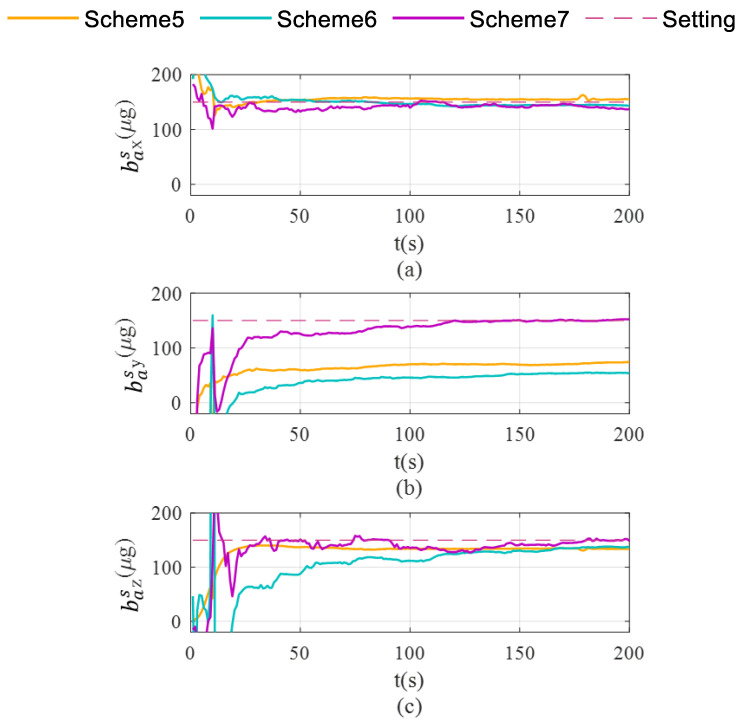
Accelerometer bias estimation in single-axis rotation with vehicle mobility condition. (**a**) accelerate bias estimation for the X-axis; (**b**) accelerate bias estimation for the Y-axis; (**c**) acceleration bias estimation for the Z-axis.

**Figure 8:**
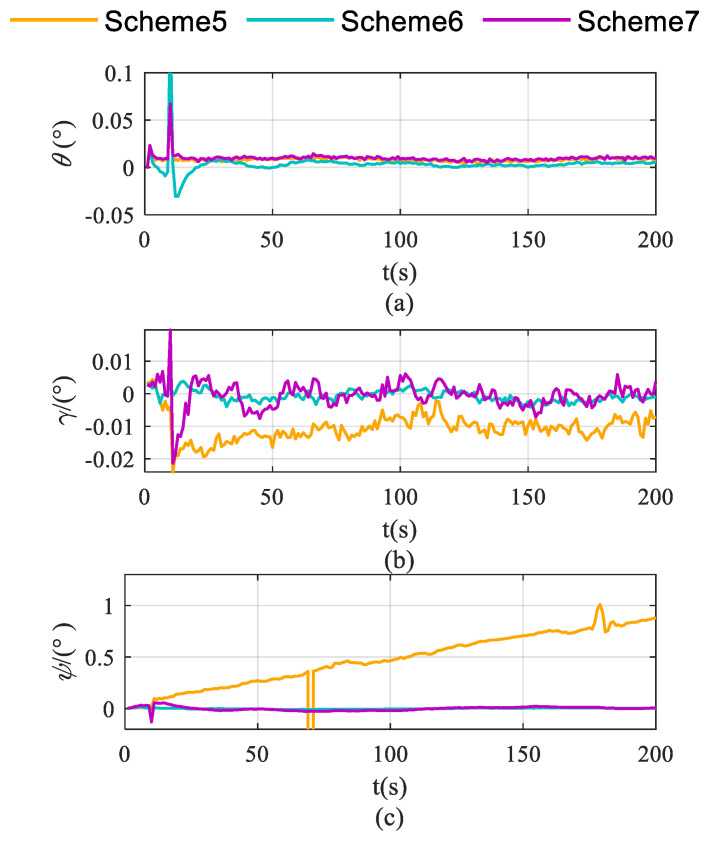
Pitch, roll and heading error accumulation characteristics in single-axis rotation with vehicle mobility condition. (**a**) for roll; (**b**) for pitch; (**c**) for heading.

**Figure 9:**
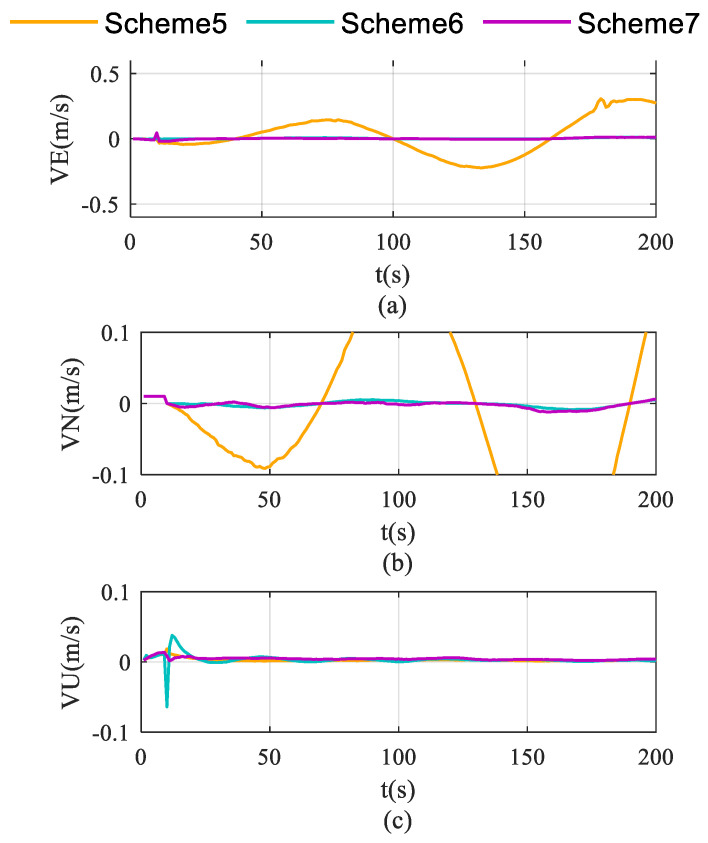
Velocity error accumulation characteristics in single-axis rotation with vehicle mobility condition. (**a**) for eastward velocity; (**b**) for northward velocity; (**c**) for vertical velocity.

Schemes are compared in terms of accelerometer bias estimation, gyroscope bias estimation, and the attitude and velocity errors shown in Figure 7, Figure 8 and Figure 9.

As shown in Figure 6c, Scheme 5 still fails to effectively correct the Z-axis gyroscope bias during uniform circular motion. In contrast, both Scheme 6 and Scheme 7 exhibit significant correction capabilities. As illustrated in Figure 7, the differential performance of the three schemes in accelerometer bias correction is clearly visualized. Scheme 5 exhibits limited correction capability for both the X-axis and Y-axis accelerometer biases. While Scheme 7 successfully corrects the biases for the X-axis and Z-axis, it fails to converge to the true value for the Y-axis (as shown in Figure 7b), yielding significantly biased results similar to those of Scheme 5. Ultimately, only Scheme 6 demonstrates comprehensive correction effectiveness across all three accelerometer axes, with its final estimates converging closely to the setting values.

Global observability analysis for the IMU single-axis rotation and vehicle mobility schemes demonstrate that Scheme 7 is constrained by the rank deficiency of its observation matrix failing to fully achieve sensor bias decoupling. Simulation results further validate this finding, demonstrating that accurate estimation of the Y-axis accelerometer bias cannot be achieved under this scheme.

As demonstrated in Figure 8a,b, Scheme 6 significantly outperforms the other two schemes in suppressing pitch and roll angle errors. This enhancement is primarily attributed to the enhanced estimation of X-axis and Y-axis gyroscopes—the key determinants of pitch and roll estimation accuracy—which effectively mitigates the error accumulation in pitch and roll.

As illustrated in Figure 8c, Scheme 5 exhibits continuously diverging heading errors, whereas both Scheme 6 and Scheme 7 effectively suppress error accumulation. This improvement originates from the enhanced observability of Z-axis bias achieved by these rotation schemes, which fundamentally mitigates the heading error divergence.

As demonstrated in Figure 9, Scheme 5 exhibits time-propagating velocity errors, whereas both rotation schemes effectively suppress these errors. Notably, Scheme 6 achieves superior error suppression, which is attributed to its significantly improved triaxial accelerometer bias estimation performance compared to the other two schemes.

The above simulation tests confirm that Scheme 5 and Scheme 6 can effectively improve the observability of the system during uniform circular motion of the vehicle, thereby achieving the separation of sensor deviations.

Simulation results indicate that the X-axis rotation scheme (corresponding to Scheme 2 under static conditions and Scheme 6 under dynamic conditions) demonstrates optimal performance among various IMU rotation modes. This scheme effectively estimates and corrects triaxial sensor biases, while significantly suppressing the accumulation of attitude and velocity errors. Its superiority stems from the fact that this rotation strategy markedly enhances the global observability of the system, achieving sufficient decoupling of sensor deviations. Consequently, the X-axis rotation scheme is established as the optimal choice for subsequent experimental design and engineering applications.

## 5. Experiment

Both theoretical analysis and simulation experiments demonstrate that the rotation scheme about the X-axis achieves optimal parameter estimation. Therefore, this scheme was adopted for vehicle data acquisition during the experimental phase.

**Figure 10:**
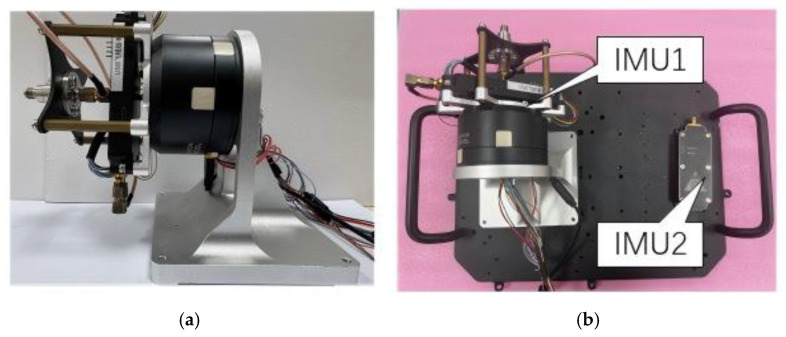
Experimental platform and designated road segment. (**a**) Rotation mechanism; (**b**) installation of the rotating system and the stationary system.

Figure 10a,b show the rotary mechanism, driven by a DJI GM6020 motor (SZ DJI Technology Co., Ltd., Shenzhen, China) and equipped with the MEMS IMU, alongside the experimental platforms. The performance parameters of the IMU used are shown in Table 3. Two experimental test cases were systematically designed to quantify the performance advantages of the rotating IMU configuration over traditional integration navigation:

The integrated navigation system employing a single fixed IMU utilizes forward velocity as an observation (single fixed IMU).

The integrated navigation system employing a single IMU rotating around the X-axis utilizes forward velocity as an observation (single rotation IMU).

The MEMS IMU modules used in our experiments are shown in Figure 10b. Each module was custom-built and integrated with two SCHA63T-K03 inertial sensor (Murata Manufacturing Co., Ltd., Nagaokakyo City, Japan) chips, with detailed gyroscope and accelerometer specifications provided in Table 3. All modules were equipped with u-blox chips, enabling data acquisition within a strictly synchronized time framework. Module 1 was responsible for collecting accelerometer and gyroscope data, while module 2 simultaneously captured motor data as well as rotary accelerometer and gyroscope data. Both modules were connected to the computer through wired connections to initiate and terminate data collection.

Before rotation began, the Y-axes of module 1 and module 2 were aligned with the vehicle’s heading direction, while the X-axis pointed to the vehicle’s right side. The X-, Y-, and Z-axes were arranged according to the right-hand rule. When rotation began, the module mounted on the rotary mechanism continuously rotated around the X-axis at a speed of 10°/s.

**Figure 11:**
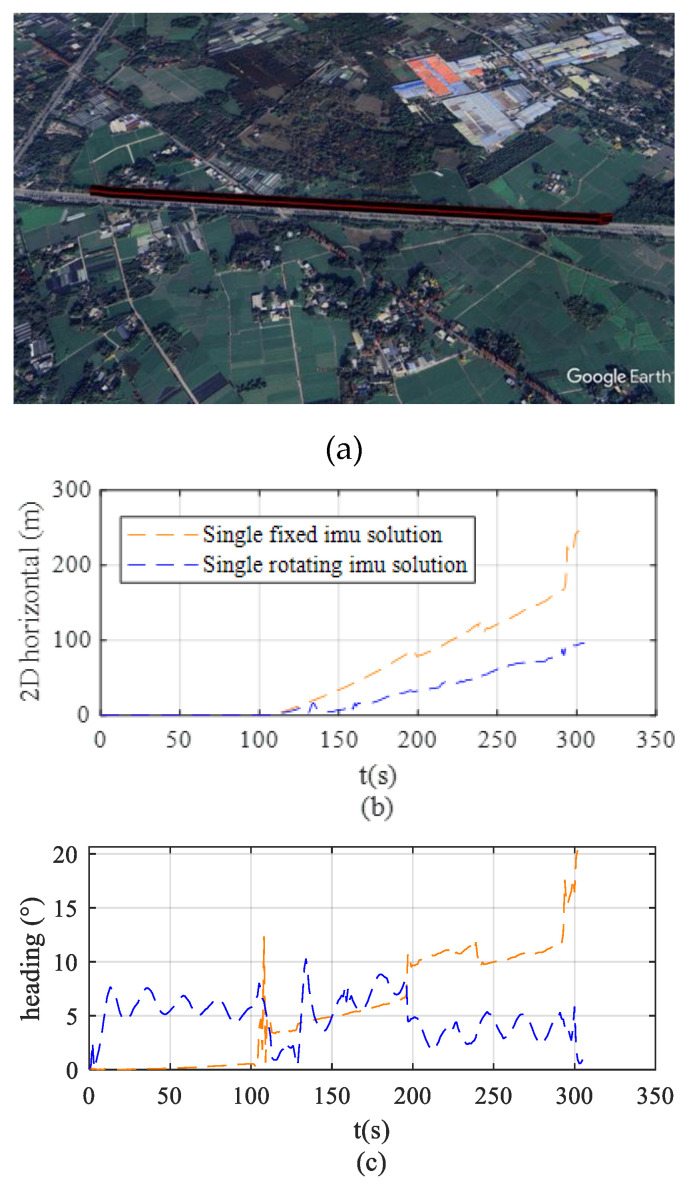
Trajectory 1 and error comparison. (**a**) Test route trajectory. (**b**) Horizontal positioning error. (**c**) Heading error.

**Figure 12:**
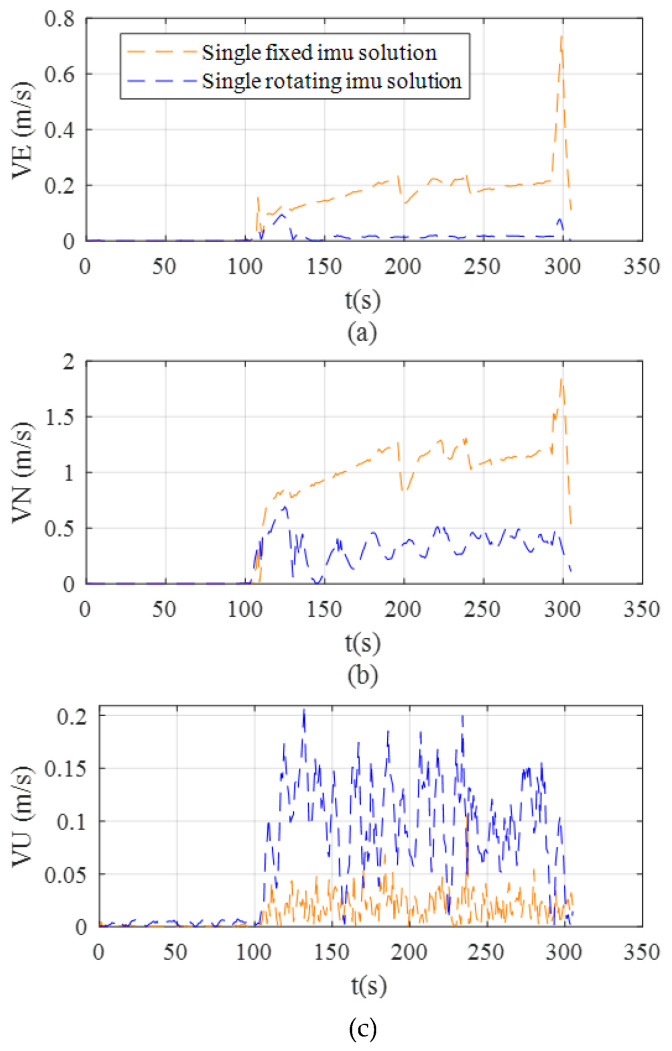
The velocity error comparison of Trajectory 1. (**a**) East velocity error. (**b**) North velocity error. (**c**) Up velocity error.

**Figure 13:**
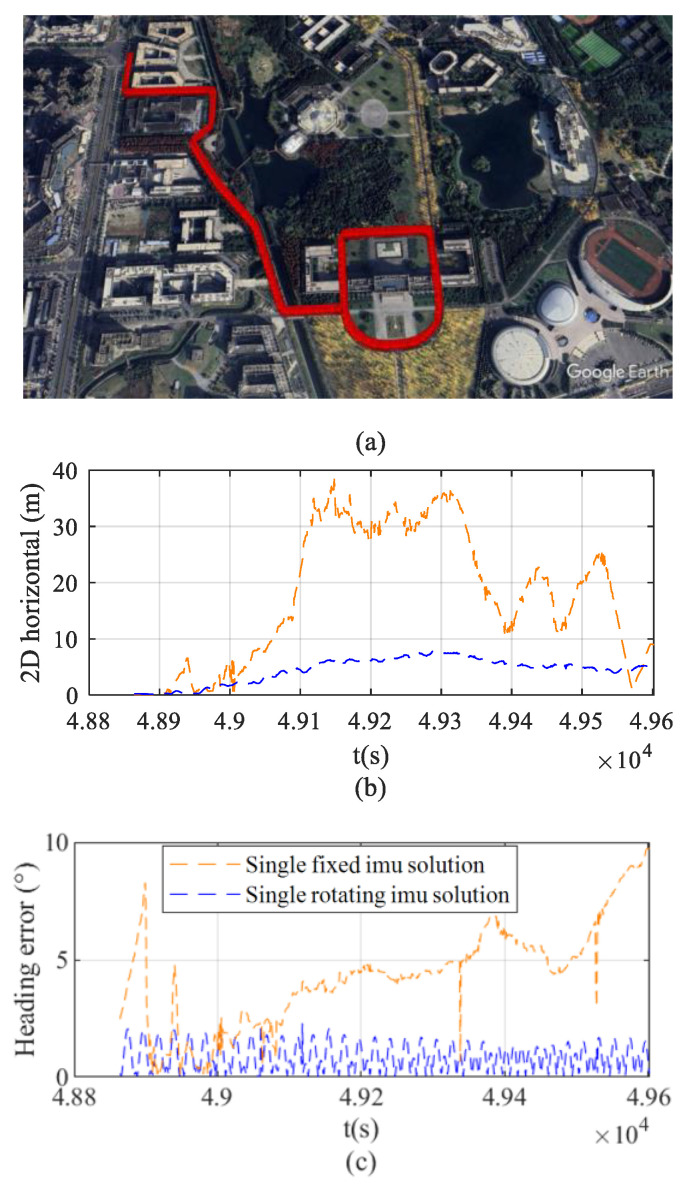
Trajectory 2 and error comparison. (**a**) Test route trajectory. (**b**) Horizontal positioning error. (**c**) Heading error.

**Figure 14:**
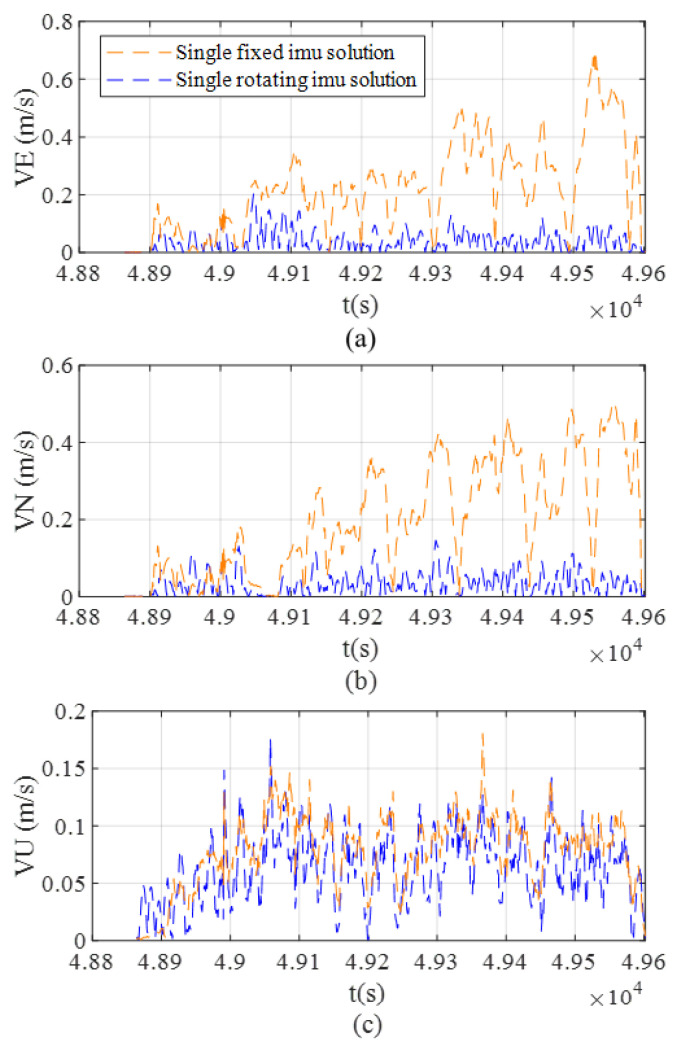
The velocity error comparison of Trajectory 2. (**a**) East velocity error. (**b**) North velocity error. (**c**) Up velocity error.

The test route incorporated both straight-line and repeated segments. This design facilitates comprehensive vehicle performance evaluation under controlled path conditions. On the unified test route, comparative experiments were conducted using the navigation systems employing a single fixed IMU, a single rotation IMU, and the high-precision Novatel’s SPAN-CPT7 (NovAtel Inc., Calgary, AB, Canada). The velocity and attitude reference was provided by the SPANCPT GPS/INS system, with a stated velocity and attitude accuracy better than 0.015 m/s and 0.06°, respectively [31]. Detailed trajectory heading and velocity comparisons are presented in Figure 11, Figure 12, Figure 13 and Figure 14.

Figure 11a shows that the field tests were conducted in Yongning Town, Wenjiang District, Chengdu City, Sichuan Province. The vehicle remains stationary for the first 110 s and travels at approximately 25 km/h in the following 196 s. The straight-line segment test is an indispensable fundamental component in the performance evaluation of integrated navigation systems, with its core advantage lying in the ability to verify the system performance of the navigation scheme in the absence of angular motion within the most simplified dynamic environment.

Figure 11b compares the evolution of horizontal positioning errors over time for vehicles using the fixed solution and the rotating solution under the same test trajectory. The results indicate that during the straight-line segment, the navigation system employing the rotating solution effectively mitigates error accumulation, resulting in significantly smaller horizontal positioning errors compared to the fixed solution. Specifically, the maximum positioning error is reduced from 248 m to 92 m, with a more stable error variation. In contrast, the horizontal positioning error of the fixed solution shows a significant divergence trend as operating time increases.

Figure 11c presents a comparison of the heading errors during the vehicle test, illustrating the variation in heading error over time for the two solutions. The experimental results show that during the vehicle’s operation phase (110 s–306 s), the heading error of the navigation system using the fixed solution accumulates significantly over time, reaching 19.5° by the end of the test. In contrast, the rotating solution effectively suppresses heading drift, with the heading error remaining stable within 5.2° after reaching a steady state. This comparison intuitively verifies the effectiveness of the rotating solution in mitigating the accumulation of heading errors.

Figure 12 compares the variation in east, north, and vertical velocity errors over time for the two solutions. The experimental results indicate that the rotating solution offers a significant advantage in velocity error suppression: after the system reaches a steady state, both the east and north errors remain at low levels (east error below 0.1 m/s; north error below 0.5 m/s), while the vertical error remains stable with no noticeable drift. In contrast, the fixed solution exhibits significant velocity error accumulation, with horizontal direction errors showing a divergent trend.

During the static phase (0–110 s), the fixed solution achieves slightly better initial suppression of heading errors compared to the rotating solution, and the rotating solution exhibits significantly greater error accumulation in vertical velocity. Based on the global observability analysis, the necessary and sufficient geometric condition for effectively decoupling sensor biases in the X-axis rotation scheme is that the projection plane of gravity’s and Earth’s rotation vectors must be orthogonal to the rotation axis. However, under actual static or low-dynamic conditions, this condition is difficult to satisfy, causing the rotational motion to result in system errors instead. As a result, the heading error and vertical velocity in the rotation solution are slightly higher than those in the fixed solution. Table 4 evaluates the 150 s–306 s data segment, during which the vehicle dynamics and sensor measurements are fully stabilized, allowing for an objective assessment of the proposed system’s steady-state performance. Specifically, the results demonstrate that under these non-maneuvering conditions, the rotating solution achieves better performance in all metrics compared to the fixed solution, with the sole exception of a larger vertical velocity error.

The field test was conducted on the Chengdu campus of the University of Electronic Science and Technology of China (UESTC), with the test route including speed bumps and typical traffic congestion as illustrated in Figure 13a. The vehicle speed was maintained at 25 km/h throughout the test. The route included repeated segments simulating uniform circular motion, designed to intuitively assess the error closure characteristics of the navigation system after extended operation. On one hand, by having the vehicle pass through the same geographic location multiple times, it is possible to verify whether the rotation scheme can preserve the observability of error states under complex road conditions, thereby providing experimental support for theoretical analysis. On the other hand, comparing the position, velocity, and attitude outputs at the same location across multiple passes enables quantitative evaluation of the repeated accuracy of the integrated navigation algorithm under identical environmental conditions, thus comprehensively validating the stability and reliability of the system.

Figure 13b compares the horizontal positioning errors of the fixed and rotating solutions over time during the repeated-segment test. The rotating scheme significantly mitigates error accumulation, reducing the maximum positioning error from 36.6 m (fixed solution) to 7.6 m, while maintaining a notably smoother error profile. Conversely, the fixed IMU configuration exhibits a pronounced divergent trend as operating time increases.

Figure 13c illustrates the temporal variation in heading error for both configurations. In the fixed setup, heading error accumulates continuously, reaching 9.5° by the test’s conclusion. In contrast, the rotating scheme effectively suppresses this drift, stabilizing the heading error within 2.2° after reaching a steady state. This clearly demonstrates the rotating solution’s efficacy in bounding heading divergence.

Furthermore, Figure 14 presents the east, north, and vertical velocity errors. The rotating scheme demonstrates superior velocity error suppression, maintaining stable errors below 0.2 m/s across all three axes. Meanwhile, the conventional fixed solution suffers from substantial error accumulation, displaying distinct divergent trends in every direction.

Table 5 demonstrates that in the presence of changes in the direction of angular velocity, the rotating solution outperforms the fixed solution.

Compared to the straight-line driving phase lacking heading changes, the single-axis rotation scheme demonstrates a more significant error suppression capability in test routes containing repeated circular maneuvers. Analyzed from the perspective of global observability theory, when the vehicle executes turning maneuvers similar to uniform circular motion, the continuous changes in its velocity vector and heading effectively break the linear dependence among the system error states. This superposition of a spatial ‘specific single-axis rotational scheme’ and temporal ‘vehicle dynamic excitation’ successfully overcomes the rank deficiency of the observation matrix. Consequently, it achieves effective decoupling of the constant biases of the triaxial gyroscopes and accelerometers, thereby substantially enhancing the observability of the system states. The experimental data further demonstrate that under the dynamic conditions stimulated by repeated segments, the rotating solution fully realizes its capability to suppress error accumulation, outperforming the conventional fixed solution in terms of positioning accuracy, heading stability, and velocity error control.

Table 5 demonstrates that when the vehicle experiences changes in the direction of angular velocity, the rotating solution outperforms the fixed system, achieving accuracy improvements of 75.3%, 62.9%, and 76% in heading error, position error, and velocity error, respectively.

In the field of autonomous driving, positioning accuracy is a key indicator of the performance. The total mileage measured by the navigation system employing the fixed solution is 2983.5 m, while that measured by the navigation system employing the rotating solution is 2926.6 m. Both values are compared against the SPAN-CPT7 reference mileage of 2921.3 m. The results demonstrate that the conventional navigation system employing a single fixed IMU yields an absolute error of 62.2 m (relative error: 2.1%), whereas the navigation system employing a single rotation IMU achieves a significantly lower absolute error of 5.3 m (relative error: 0.18%).

Experimental verification shows that the rotation scheme around the X-axis significantly suppresses the accumulation error of the integrated navigation system. Quantitative analysis of straight-line and repeated road segments, based on mean, extremum, and standard deviation indicators, further confirms that the integrated system using the proposed scheme achieves substantial improvements in horizontal positioning accuracy, speed stability, and heading maintenance ability.

## 6. Conclusions

This paper presents a novel integrated navigation system utilizing an independent uniform rotation mechanism to overcome the inherent accuracy limitations of micro-electromechanical inertial sensors in environments without global navigation satellite system signals. Through comprehensive global observability analysis, the research establishes that rotating the inertial measurement unit around the X-axis optimally decouples sensor biases and minimizes long-term error accumulation. Both simulation models and field experiments validate the theoretical findings, demonstrating that this specific rotation scheme significantly outperforms stationary configurations. Notably, during complex maneuvers involving angular velocity changes, the proposed architecture reduced heading, position, and velocity errors by 75.3%, 62.9%, and 76% respectively. These results confirm the feasibility, initial performance improvements, and superior parameter estimation capabilities of the X-axis rotation scheme, offering a highly adaptable and scalable solution for high-precision autonomous vehicle navigation.

In practical engineering applications, MEMS IMUs typically undergo rigorous factory calibration to compensate for deterministic constant biases. Consequently, the system’s performance during operation is primarily affected by random drift. It is worth noting that these slowly varying random errors effectively manifest as a fixed bias at any given instant. Leveraging this physical characteristic, this paper introduces a specific rotation scheme within the integrated navigation framework. This scheme enables the real-time transformation of these transiently fixed random drifts into observable biases. This mechanism provides a theoretical guarantee that the performance gains achieved by this approach remain highly robust, even when the sensor’s random drift fluctuates within the nominal uncertainty levels typical of MEMS devices.

While the current study successfully demonstrates the feasibility of the X-axis rotation scheme, the validation remains a preliminary proof-of-concept. Empirically, the field tests were limited to a single-run trial under a constant rotation speed of 10°/s and an exclusive focus on the X-axis configuration; the lack of repeated trials to statistically verify performance consistency constitutes a notable limitation. Furthermore, in practical deployments, non-ideal factors such as installation misalignments, encoder errors, unmodeled kinematics, and the choice of rotation speed itself will inevitably introduce errors and long-term error accumulation. Consequently, establishing a more comprehensive experimental framework with repeated statistical trials, systematically investigating the impact of varying rotation speeds, and conducting rigorous quantitative evaluations of these practical engineering impacts represent essential and highly valuable directions for our future research.

## Figures and Tables

**Table 1:** List of solutions for vehicle stationary conditions.

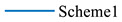	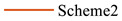	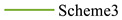	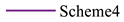
Scheme 1: a test with non-rotating MEMS IMU	Scheme 2: a test with the MEMS IMU rotating around the X-axis	Scheme 3: a test with the MEMS IMU rotating around the Y-axis	Scheme 4: a test with the MEMS IMU rotating around the Z-axis

**Table 2:** List of solutions for vehicle mobility conditions.

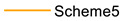	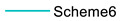	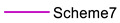
Scheme 5: a test with vehicle mobility	Scheme 6: a test with the MEMS IMU rotating around the X-axis and vehicle mobility	Scheme 7: a test with the MEMS IMU rotating around the Y-axis and vehicle mobility

**Table 3:** Inertial navigation device parameters.

IMU	Gyro Bias (°/s)	ARW * ( ${}^{\circ}/\sqrt{h}$	Bias Instability (°/h)	Acc. * Bias (mg)	VRW * ($mm/s/\sqrt{h}$)	Acc. Instability (ug)
SCHA63T-K03	≤|10.8|	0.07	1.11	≤|13.5|	35	12.2

* ARW denotes the angle random walk; Acc. denotes the accelerometer; VRW denotes the velocity random walk.

**Table 4:** RMS errors of heading, position, and velocity for the fixed IMU solution and the rotating IMU solution during the 150 s–306 s interval of Trajectory 1.

	Heading (°)	Position (m)	Horizontal Velocity (m/s)	Vertical Velocity (m/s)
Fixed IMU solution	9.71	121.73	1.12	0.0258
Rotating IMU solution	4.66	54.33	0.37	0.1088

**Table 5:** RMS errors of heading, position, and velocity for the fixed IMU solution and the rotating IMU solution during the 8–12 min interval of Trajectory 2.

	Heading (°)	Position (m)	Horizontal Velocity (m/s)	Vertical Velocity (m/s)
Fixed IMU solution	3.9	20.8	0.36	0.07
Rotating IMU solution	0.96	7.7	0.071	0.05

## Data Availability

The data presented in this study are available on request from the corresponding author. The data are not publicly available due to commercial restrictions.
